# Reassessing BMI thresholds for obesity: a population-based study in Turkish adults

**DOI:** 10.1038/s41366-026-02142-y

**Published:** 2026-06-26

**Authors:** Semiha Zeynep Özsaydı, Hasan Durmuş, Leyla Tekdemir, Seçkin Özsaydı, Arda Borlu, Neslihan Öner

**Affiliations:** 1 Health Directorate of Kayseri, Kayseri, Turkey; 2 Department of Public Health, School of Medicine, Erciyes University, Kayseri, Turkey; 3 Erciyes University, Faculty of Health Sciences, Department of Nutrition and Dietetics, Kayseri, Turkey

**Keywords:** Risk factors, Body mass index

## Abstract

**Background:**

The global prevalence of obesity has been steadily increasing, posing major public health challenges. Current universal BMI cut-off points may not accurately reflect obesity risk across different ethnicities, including the Turkish population.

**Objective:**

This study aimed to evaluate the relationship between body mass index (BMI) and body fat percentage (BF%) and determine appropriate BMI cut-off points for diagnosing obesity in Turkish adults.

**Methods:**

A cross-sectional study was conducted among Turkish individuals aged 18–65 in four major districts of Kayseri. Anthropometric measurements were obtained, including height, weight, waist circumference, and hip circumference. Body composition was assessed using bioelectrical impedance analysis (BIA). ROC (Receiver Operating Characteristic) curve analysis was performed to identify optimal BMI cut-off points for predicting obesity based on BF%. Pearson correlation analysis evaluated the relationship between BMI and BF%.

**Results:**

The participants had a mean age of 39.9 ± 13.3 years, and 51.6% were female. A strong positive correlation between BMI and BF% was found for both women (*r* = 0.86, *p* < 0.001) and men (*r* = 0.68, *p* < 0.001). The optimal BMI cut-off point for detecting obesity was 28.76 kg/m² for the total sample, 28.80 kg/m² for women, and 28.76 kg/m² for men. Using these new thresholds, the obesity prevalence increased from 27.7% to 33.6%. The AUC values indicated excellent discriminative power for women (AUC = 0.98) and good discriminative power for men (AUC = 0.91).

**Conclusion:**

The current universal BMI cut-off points (≥30 kg/m²) may underestimate obesity prevalence in the Turkish population. Establishing national and gender-specific BMI thresholds could improve the accuracy of obesity diagnosis and support more effective public health interventions targeting chronic non-communicable diseases.

## Introduction

Obesity, characterized by an excessive accumulation of adipose tissue, has become an increasingly prevalent global public health issue and constitutes a major risk factor for numerous chronic conditions, including cardiovascular diseases, type 2 diabetes, hypertension, and various types of cancer [1, 2]. The World Obesity Federation defines obesity as a chronic, recurrent, progressive disease process [3]. In 2019, overweight- and obesity-related non-communicable diseases (NCDs) were estimated to have caused approximately five million deaths globally [1].

Obesity can be assessed using advanced imaging techniques such as dual-energy X-ray absorptiometry (DEXA) and magnetic resonance imaging (MRI) [4]. Dual-energy X-ray absorptiometry (DEXA), regarded as a reference method for body composition, is impractical for large-scale population surveys due to its high cost, limited accessibility, and operational complexities. Consequently, easily accessible, low-cost, and rapid assessment methods are preferred. Among these, bioelectrical impedance analysis (BIA) emerges as a practical alternative for body composition estimation, owing to its high precision (repeatability) and ease of use DEXA [5]. Furthermore, simple anthropometric indices such as body mass index (BMI), waist circumference (WC), and waist-to-hip ratio (WHR) are routinely utilized for the evaluation of obesity in large populations due to their ease of application and cost-effectiveness [4].

The most commonly utilized metric for diagnosing obesity is body mass index (BMI), calculated as body weight (kg) divided by the square of height (m²). Based on the World Health Organization (WHO) classification, BMI values of 18.5–24.9 kg/m² are regarded as normal, 25–29.9 kg/m² as overweight, and values equal to or exceeding 30 kg/m² as indicative of obesity among adults [4, 6]. However, the biological and epidemiological appropriateness of these universal cut-off points for all populations has been considerably debated over the last 25 years. Although BMI offers the advantages of practicality and widespread availability, it does not account for body composition or fat distribution, which constitutes a significant limitation. While BMI is correlated with body fat percentage (BF%), it primarily reflects excess body weight rather than excess adiposity [7]. On the other hand, to more accurately evaluate the health impacts of obesity, it has been emphasized that excess fat accumulation should be considered the primary determinant in the diagnosis of obesity. Current BMI-based assessments may result in underestimating or overestimating obesity prevalence, potentially compromising the effectiveness of evidence-based decision-making in health service planning and public health policy development. Consequently, the literature highlights the necessity of validating excess adiposity through accurate methods and utilizing appropriate cut-off points for age, gender, and ethnicity [8]. Excess adiposity refers to the accumulation of adipose tissue beyond physiological needs, which drives the metabolic and cardiovascular complications of obesity. A major limitation of BMI is its inability to distinguish between fat mass and fat-free mass. Consequently, physically active individuals with high muscle mass may be misclassified as overweight or obese despite having low body fat, while individuals with a normal BMI might harbor excess visceral adiposity (normal-weight obesity), which carries substantial health risks [9]. For example, evidence suggests that Asians are at elevated risk for diabetes and cardiovascular diseases even at BMI values lower than 25 kg/m² and display greater abdominal adiposity and BF% relative to their Western counterparts at comparable BMI levels [10, 11]. Accordingly, the WHO has suggested revised BMI cut-off points for the Asia-Pacific population, designating individuals with a BMI ≥ 23 kg/m² as overweight as overweight and those with a BMI ≥ 27.5 kg/m² as obese [12]. Evidence from a study in the Omani population indicates that BMI thresholds should be lower than those applied to Western populations, given that metabolic risk factors escalate at comparatively lower BMI values among Omanis [13]. In contrast, some evidence indicates that individuals of European ancestry with a BMI exceeding 30 kg/m² may exhibit lower body fat levels than expected and may not face an increased risk of morbidity or mortality [8]. A growing body of evidence underscores that ethnic variations are critical determinants in the diagnosis of obesity, advocating for BMI cut-off points that are closely associated with BF%. Supporting this, a comprehensive meta-analysis of 32 studies revealed substantial differences in the association between BF% and BMI among diverse ethnic populations [14]. The findings underscore the importance of identifying population-specific BMI thresholds for obesity diagnosis, particularly in Türkiye, which exhibits considerable genetic, environmental, and cultural diversity. Nevertheless, research addressing this issue within the Turkish population remains insufficient [15]. This study aimed to assess the association between BF% and BMI in the target population and identify valid BMI thresholds that can be utilized to diagnose obesity in Türkiye with greater accuracy.

## Materials and methods

### Study design

This cross-sectional and descriptive study was conducted among Turkish individuals aged 18–65 years residing in the four central districts—Kocasinan, Melikgazi, Talas, and Hacılar—that comprise the Kayseri provincial center. These districts account for ~80% of the total population of Kayseri province, and the study population consisted of adults living within these districts. The sample size for the study was calculated using EpiInfo 7.2 software, with a 5% absolute margin of error and a 95% confidence level, based on the 21.1% obesity prevalence reported among individuals aged ≥15 years according to the Turkish Health Statistics Yearbook 2022. As a result of these calculations, the minimum required sample size was determined to be *n* = 256 individuals. The population sizes for the 18–25, 26–35, 36–45, 46–55, and 56–65 age groups were determined separately for both genders, and the total sample size was proportionally distributed across the age groups based on these data [16]. Thus, the number of individuals to be included from each age group was calculated to reflect the demographic structure of the province accurately. Participants were selected from individuals listed in the national Public Health Management System (PHMS) database of Türkiye using simple random sampling, weighted by population size and age distribution. Pregnant women, individuals actively engaged in bodybuilding activities, and those with pacemakers were excluded from the study. Data collection was conducted at the Mustafa Kızıklı Healthy Life Center, located in the Kocasinan district of Kayseri, under the supervision of a dietician. Written informed consent was obtained from all participants prior to their inclusion in the study.

### Data collection

Anthropometric measurements of the participants were obtained using a bioelectrical impedance analysis (BIA) device, a stadiometer, and a tape measure. For each participant, all anthropometric and body composition measurements were conducted during a single morning session, under fasting conditions, with participants wearing light clothing, without shoes, and free of any metal objects. Each participant underwent the following anthropometric assessments:

- Weight (kg)
- Height (cm)
- Body fat percentage (BF%)
- Waist circumference (cm)
- Hip circumference (cm)

Weight and BF% were measured using BIA. Measurements were performed with a Tanita Innerscan Dual RD-545 BIA analyzer (Tanita Corporation, Tokyo, Japan). This dual-frequency BIA device has been recently validated against dual-energy X-ray absorptiometry (DXA), demonstrating high accuracy, acceptable body composition estimates, and high test-retest reliability for body fat percentage and muscle mass assessments [17]. The device protocols maintained proper positioning and hydration levels during the measurements. The participants’ height was measured to the nearest 0.1 cm using a stadiometer, with participants standing upright and ensuring that four points—the heel, calf, hip, and shoulder—were in contact with the vertical board and with shoes removed. Waist and hip circumferences were measured to the nearest 0.1 cm using an inelastic tape. Waist circumference (WC) was measured at the midpoint between the lower margin of the rib cage and the iliac crest following a full expiration [18]. Hip circumference (HC) was measured at the point of maximum protrusion of the hips, with participants standing upright, feet together, and in a relaxed posture [19].

Body Mass Index (BMI), Waist-to-Height Ratio (WHtR), and Waist-to-Hip Ratio (WHR) were calculated using the following formulas based on the anthropometric measurement values obtained from the participants as described above:

- BMI: Weight (kg/m²)
- WHR: Waist circumference (cm)/hip circumference (cm)
- WHtR: Waist circumference (cm)/height (cm)

In this study, obesity was classified based on BF% thresholds that vary according to age and gender. This classification was derived from the BMI- and age-specific reference ranges proposed by Gallagher et al. [20]. Based on the following thresholds:

For males:

- 18–39 years: BF% ≥ 25
- 40–59 years: BF% ≥ 28
- 60–79 years: BF% ≥ 30
  For females:
- 18–39 years: BF% ≥ 39
- 40–59 years: BF% ≥ 40
- 60–79 years: BF% ≥ 42

Individuals were categorized as “obese” or “not obese” according to these threshold values, with obesity status treated as a binary variable.

### Statistical analysis

Data analysis was conducted using the SPSS 21.0 software and the Python 3.11 programming language. Descriptive statistics for continuous variables were presented as mean ± standard deviation (SD), while categorical variables were summarized using frequencies and percentages. Pandas, an open-source data analysis tool used in the Python programming language, was utilized for data cleaning, transformation, and calculations; Sci-Kit Learn was utilized for ROC curve generation and classification analysis; and the matplotlib and seaborn libraries were employed for graph creation. In this study, the ability of BMI to discriminate against obesity, as defined by BF% thresholds classified according to age and gender, was evaluated using ROC (Receiver Operating Characteristic) analysis. Sensitivity and specificity values were calculated based on the ROC curves. The resulting ROC curves, AUC (Area Under the Curve) values, and optimal cut-off points were presented graphically. The relationship between BMI and BF% measured by BIA was assessed using Pearson correlation analysis. To account for physiological differences in adiposity distribution, this analysis was performed separately for men and women. The gender-specific regression lines and correlations were generated in R 4.3.2 utilizing the ggstatsplot package [21]. A correlation graph, a scatter plot illustrating the linear relationship between variables, marginal histograms, the Pearson correlation coefficient, the *p*-value, and the confidence interval were reported.

## Results

The mean age of the participants was 39.9 ± 13.3 years. Of the group, 51.6% (*n* = 132) were female. The mean height was 167.15 ± 9.66 cm, and the mean weight was 75.98 ± 16.05 kg. Selected anthropometric measurements obtained from the participants are presented in Table 1.

**Table 1:** Anthropometric characteristics of participants by gender.

Variables	Total (*n* = 256) Mean ± SD	Male (*n* = 124) Mean ± SD	Female (*n* = 132) Mean ± SD
Height (cm)	167.15 ± 9.66	174.89 ± 6.35	159.87 ± 5.81
Weight (kg)	75.98 ± 16.05	83.60 ± 15.18	68.83 ± 13.36
BMI (kg/ m²)	27.23 ± 5.04	27.48 ± 4.81	26.98 ± 5.26
Waist circumference (cm)	92.82 ± 14.78	98.73 ± 14.08	87.27 ± 13.23
Hip circumference (cm)	104.88 ± 9.13	105.26 ± 9.93	104.53 ± 8.33
Body fat percent (BF%)	30.74 ± 8.64	25.54 ± 7.18	35.62 ± 6.88
WHR	0.88 ± 0.08	0.93 ± 0.05	0.83 ± 0.07
WHtR	0.55 ± 0.08	0.56 ± 0.08	0.54 ± 0.08

The classification of the participants’ obesity status according to the BMI thresholds recommended by the WHO is presented in Table 2.

**Table 2:** Obesity classification of participants according to BMI thresholds.

Obesity classification	Total (*n* = 256) *n* (%)	Male (*n* = 124) *n* (%)	Female (*n* = 132) *n* (%)
Non-obese (BMI < 30 kg/ m²)	185 (72.3)	89 (71.8)	96 (72.7)
Obese (BMI ≥ 30 kg/ m²)	71 (27.7)	35 (28.2)	36 (27.3)

While the prevalence of obesity based on BMI was 27.7% among all participants, the prevalence of obesity based on BF% thresholds according to the Gallagher classification, which varies by age and gender was 33.6%. The classification of the participants’ obesity status according to BF% thresholds varying by age and gender is presented in Table 3.

**Table 3:** Classification of participants by obesity status using age- and sex-specific BF% cut-off values.

Male (*n* = 124) *n* (%)		Female (*n* = 132) *n* (%)	
18–39 years		18–39 years	
Without-obesity (BF < 25)	34(27.4)	Without-obesity (BF < 39)	52(39.3)
With obesity (BF ≥ 25)	26(20.9)	With obesity (BF ≥ 39)	12(9.0)
40–59 years		40–59 years	
Without-obesity (BF < 28)	32(25.8)	Without-obesity (BF < 40)	33(25.3)
With obesity (BF ≥ 28)	18(14.5)	With obesity (BF ≥ 40)	23(17.4)
60–79 years		60–79 years	
Without-obesity (BF < 30)	12(9.6)	Without-obesity (BF < 42)	7(5.3)
With obesity (BF ≥ 30)	2(1.6)	With obesity (BF ≥ 42)	5(3.8)

**Fig. 1:**
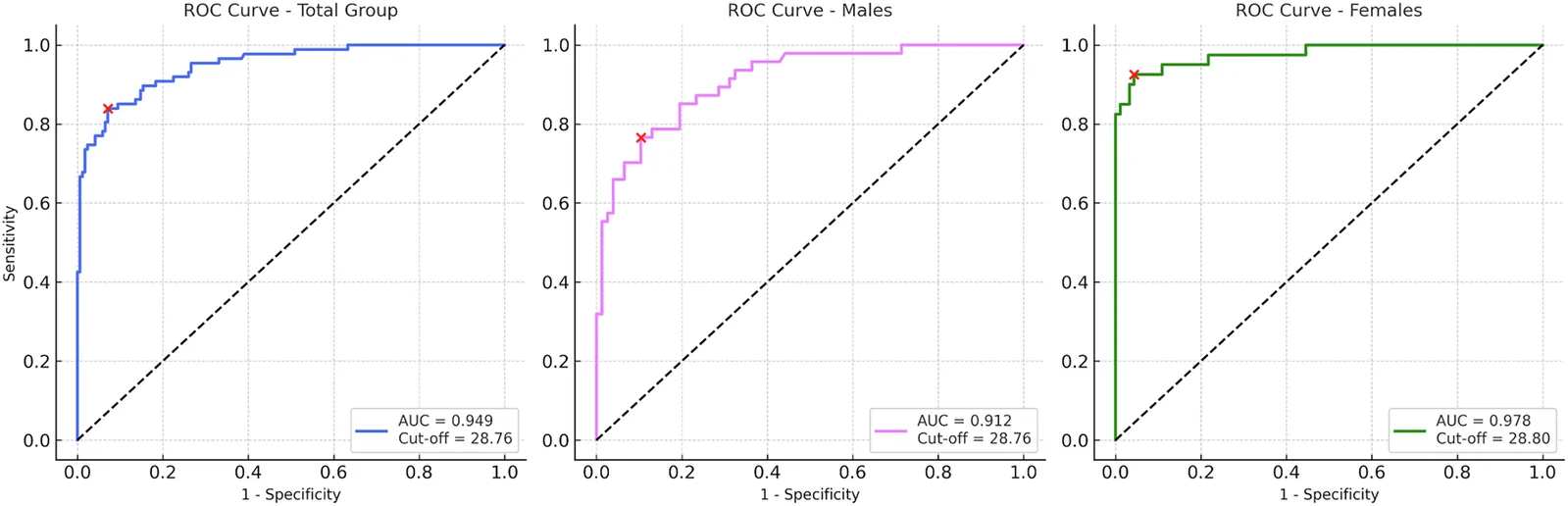
Receiver Operating Characteristic (ROC) curves demonstrating the diagnostic performance of Body Mass Index (BMI) for predicting obesity based on Body Fat Percentage (BF%) thresholds. The panels display the curves for the total group, males, and females, including Area Under the Curve (AUC) values and optimal cut-off points.

The obesity status of the participants and the newly evaluated BMI cut-off points are presented in Fig. 1. The AUC value, indicating the ability of BMI to discriminate against people with obesity individuals, was calculated as 0.95. The optimal cut-off value was determined to be 28.76 kg/m², with a sensitivity of 83.7% and a specificity of 91.8% at this threshold. These findings demonstrate that BMI exhibits a good level of performance in distinguishing obesity among all participants.

When evaluated by gender, the AUC value for females was found to be 0.98, with an optimal cut-off point of 28.80 kg/m², a sensitivity of 92.5%, and a specificity of 95.7%. In males, the AUC value was calculated as 0.91, with a cut-off point of 28.76 kg/m², a sensitivity of 76.1%, and a specificity of 88.5%. The diagnostic accuracy of BMI was also high within gender subgroups; an AUC value above 0.90 in the female group was interpreted as indicating “excellent” discriminatory power, while the male group was considered to reflect a “good” level of diagnostic performance. When the newly determined BMI cut-off points were applied, the prevalence of obesity in the overall group increased from 27.7% to 33.6%. The classification of the obesity status of the participants according to the newly obtained BMI cut-off points is presented in Table 4. The detailed diagnostic performance metrics of the optimal BMI cut-off points, including sensitivity, specificity, positive predictive value (PPV), and negative predictive value (NPV) calculated separately for both genders, are presented in Table 5.

**Table 4:** Participant classification according to the newly determined BMI cut-off point (28.80 kg/m²).

Total (*n* = 256) *n* (%)		Male (*n* = 124) *n* (%)		Female (*n* = 132) *n* (%)	
Without obesity (BMI < 28.8 kg/m²)	170 (66.4)	Without obesity (BMI < 28.8 kg/m²)	80 (64.5)	Without obesity (BMI < 28.8 kg/m²)	90 (68.2)
With obesity (BMI ≥ 28.8 kg/m²)	86 (33.6)	With obesity (BMI ≥ 28.8 kg/m²)	44 (35.5)	With obesity (BMI ≥ 28.8 kg/m²)	42 (31.8)

**Table 5:** Diagnostic performance of optimal BMI cut-off points for predicting obesity by gender.

Group	Optimal BMI cut-off (kg/m²)	Sensitivity (%)	Specificity (%)	Positive predictive value (PPV) (%)	Negative predictive value (NPV) (%)
Men (*n* = 124)	28.76	76.1	88.5	81.8	86.3
Women (*n* = 132)	28.80	92.5	95.7	88.1	96.7

**Fig. 2:**
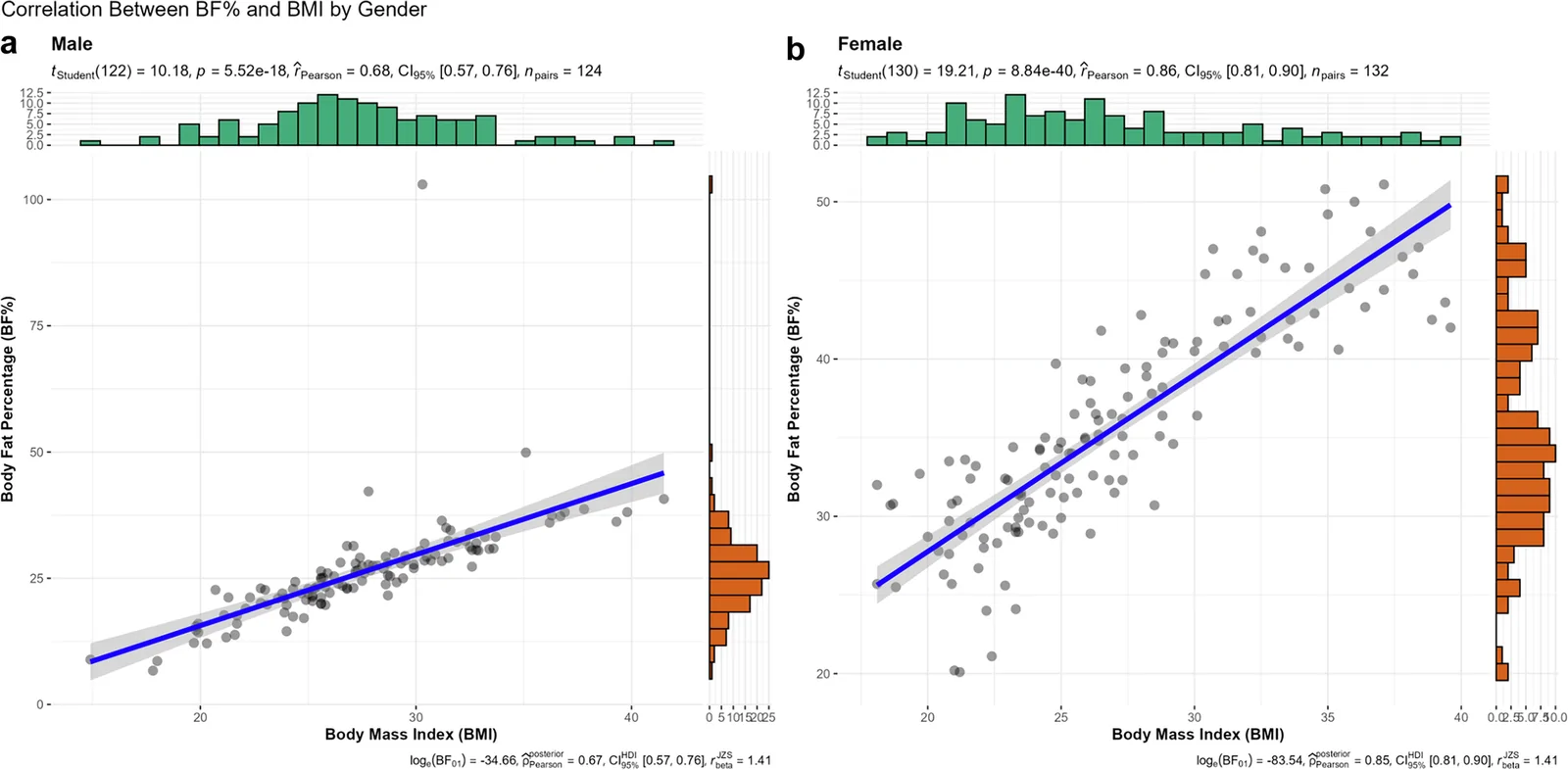
Correlation between BMI and Body Fat Percentage by Gender. Scatter plots with marginal histograms illustrating the Pearson correlation between Body Mass Index (BMI) and Body Fat Percentage (BF%) evaluated by Bioelectrical Impedance Analysis (BIA) in (**a**) male and (**b**) female participants.

The relationship between BMI and BF% measured by BIA was analyzed using Pearson correlation analysis separately for both sexes (Fig. 2). A statistically significant and strong positive correlation was observed in women (*r* = 0.86, *p* < 0.001). In men, the correlation was also significant and moderately strong (*r* = 0.68, *p* < 0.001). These results emphasize the sex-specific differences in the relationship between body weight and adiposity.

## Discussion

The global prevalence of obesity more than doubled between 1990 and 2022 [1]. In Türkiye, the prevalence of obesity, a major risk factor for non-communicable diseases, is higher than the average of OECD countries and has been steadily increasing over the years [22]. In 2016, Türkiye had the highest prevalence of obesity (29.5%) among the countries in the WHO European Region [23]. The prevalence of obesity observed in our study is consistent with findings reported in the literature. This suggests that the risk of morbidity and mortality due to obesity-related diseases is increasing within the Turkish population. It is anticipated that establishing accurate BMI cut-off points specific to the Turkish population will play a critical role in combating obesity-related diseases by enabling the accurate diagnosis of obesity and the correct identification of at-risk groups, thereby reducing future health and economic burdens. Türkiye spans Asia and Europe, with the majority of its population (85.9%) residing in the Asian part of the country [16]. However, there has been considerable debate regarding the appropriateness of applying cut-off values derived from Western populations to other groups, such as Asian populations, when assessing obesity [24]. The debates surrounding these issues are rooted in recognized ethnic differences in BF% and associated obesity-related health risks. In response, the WHO and the National Institute for Health and Care Excellence (NICE) have recommended lowering the BMI cut-off point to 27.5 kg/m² for identifying obesity in South Asian and Chinese populations, alongside the development of culturally appropriate interventions to address this concern [12, 25]. A UK cohort study found that South Asians experienced an equivalent risk of developing type 2 diabetes as White individuals at a BMI of 23.9 kg/m², compared to a BMI of 30.0 kg/m² for White individuals [26]. In a study conducted in Iran, a neighboring country of Türkiye and part of the Asian region, the appropriate BMI cut-off value was determined to be 27.70 kg/m² [27]. In a study conducted on 202 Turkish women investigating appropriate anthropometric methods and cut-off points for predicting metabolic syndrome (MS), a new BMI cut-off value of 27.7 kg/m² was identified [28]. Our study findings indicate that the optimal BMI threshold for detecting obesity in the Turkish population is 28.76 kg/m², which differs from the standard reference cut-off. This suggests a need to reconsider the use of the current BMI cut-off to diagnose obesity in this population, as it may overlook individuals classified as obese according to the newly identified threshold. Our newly identified threshold is consistent with regional literatüre and underscore the importance of developing culturally appropriate interventions to address non-communicable chronic diseases effectively.

Chronic non-communicable diseases have seen a rise in incidence both globally and within Türkiye. In response, global and national health strategies aim to curb the increase in diabetes and obesity while also working to achieve a 25% relative reduction in premature deaths caused by cardiovascular diseases and diabetes [29]. These targets highlight the importance of the accurate and timely identification of obesity for public health efforts. Our study found that the prevalence of obesity was 27.7% according to the universal BMI reference values recommended by the WHO but increased to 33.6% when the newly determined BMI cut-off points were applied. This is consistent with another hospital-based study in Türkiye, which reported the prevalence of obesity rose to 33.9% when recalculated BMI thresholds based on BF% were used, suggesting the standard BMI reference value underestimated the true prevalence of obesity [15]. The observation that the current BMI cut-off value yields a lower prevalence of obesity indicates the inadequacy of using universal reference values for diagnosing obesity in the Turkish population. Historically, BMI was primarily developed to assess malnutrition and underweight populations rather than to quantify obesity-related health risks. As highlighted by large-scale systematic reviews, including those published in JAMA, standard BMI categories often fail to capture the nuances of excess adiposity, and strict numerical cut-offs may not accurately reflect true all-cause mortality or morbidity risks across diverse populations [30, 31]. This finding suggests that a larger segment of the population should be targeted in efforts to combat obesity, a major modifiable risk factor for numerous non-communicable chronic diseases. Otherwise, individuals with obesity may remain unaware of their condition and fail to seek or receive necessary preventive health interventions.

While searching for appropriate BMI cut-off points in the literature, it has been observed that differences may exist at the population level and by gender. For example, in a study conducted in Iran, the appropriate BMI cut-off value was found to be 27.7 kg/m² for women and slightly lower, at 27.3 kg/m², for men [27]. In a study conducted among Ethiopian adults, the appropriate BMI cut-off value was found to be higher for women (24.5 kg/m²) compared to men (22.2 kg/m²) [32]. In our study, the newly determined cut-off value for women was higher than that for men, although both values were lower than the current universal reference, consistent with findings observed in Asian populations. The distribution of adipose tissue in the body differs between sexes, with the android type being more predominant in men and the gynaecoid type more prevalent in women. It is well established that abdominal obesity carries a higher risk for certain chronic diseases associated with metabolic alterations, such as type 2 diabetes mellitus and cardiovascular diseases [33]. Although the optimal BMI cut-off values were virtually identical for both sexes (~28.8 kg/m²), the diagnostic performance of BMI varied significantly between genders. Specifically, the sensitivity of this BMI threshold was much higher in women (92.5%) compared to men (76.1%). The distribution of adipose tissue differs between sexes, with the android (central) type being more predominant in men and the gynoid type more prevalent in women. As highlighted by the recent 2025 NICE guidelines, BMI is not a direct measure of central adiposity and must be interpreted with caution. This physiological difference may explain why BMI alone is a less accurate predictor of excess adiposity in men, underscoring the necessity of using supplementary measures like waist-to-height ratio to better reflect health risks in the male population [34].

In a comprehensive meta-analysis conducted by Deurenberg et al., it was observed that the relationship between BF% and BMI cut-off points varied across different ethnic groups [14]. Our study found a statistically significant and strong correlation between BMI and BF%. This finding is consistent with the results reported by Jahanlou et al. [27] and Bozkirli et al. [15]. While BMI is a good predictor of BF%, it is well-established that BF% is a more important risk factor than body weight for the development of chronic diseases [14]. Our findings suggest that determining accurate cut-off points will make predictions more robust, enabling more precise and appropriate measures against risk factors. A limitation of this study is the reliance on Bioelectrical Impedance Analysis (BIA) rather than reference methods such as Dual-energy X-ray Absorptiometry (DXA) or Magnetic Resonance Imaging (MRI) for body composition assessment in our specific cohort. However, the dual-frequency BIA device used in this study (Tanita RD-545) has been previously validated against DXA and shown to be highly accurate and reliable. Furthermore, while the Gallagher classification is universally recognized for defining healthy body fat ranges, the absence of population-specific BF% normative reference standards for Turkish adults constitutes an additional limitation when establishing these new cut-off points.

To the best of our knowledge, there are only a limited number of studies on this subject among the Turkish population. However, existing studies either utilized smaller sample sizes without reference methods or were hospital-based, thus inadequate in representing the general population. In this context, our study is well-positioned to contribute to future research by helping to establish a national BMI cut-off point for Türkiye, thereby serving as a guide for future interventions and public health measures.

## Conclusion

In our study, a significant and strong relationship was observed between BMI and BF%, and BMI was found to be an effective tool for predicting obesity. However, the findings suggest that the current universal BMI cut-off points are not fully applicable to the Turkish population, potentially leading to an underestimation of obesity in certain population segments. Gender-specific analyses further revealed that the optimal BMI cut-off value was higher for women than men.

- Although gender-specific analyses were performed, the optimal BMI cut-off values were nearly identical for both men and women (28.76 and 28.80 kg/m², respectively). Therefore, a unified population-specific BMI threshold of ~28.8 kg/m² can be recommended for Turkish adults regardless of gender.
- Studies involving larger, nationally representative samples are needed to strengthen this field’s data and support the revision and updating of public health strategies.

## Data Availability

The datasets generated and/or analyzed during the current study are not publicly available due to participant confidentiality and ethical restrictions but are available from the corresponding author on reasonable request.
